# Inferring genetic interactions via a nonlinear model and an optimization algorithm

**DOI:** 10.1186/1752-0509-4-16

**Published:** 2010-02-26

**Authors:** Chung-Ming Chen, Chih Lee, Cheng-Long Chuang, Chia-Chang Wang, Grace S Shieh

**Affiliations:** 1 Institute of Biomedical Engineering, National Taiwan University, No 1, Sec 4, Roosevelt Road, Taipei, 106, Taiwan; 2 Institute of Statistical Science, Academia Sinica, No 128, Sec 2, Academia Road, Taipei 115, Taiwan

## Abstract

**Background:**

Biochemical pathways are gradually becoming recognized as central to complex human diseases and recently genetic/transcriptional interactions have been shown to be able to predict partial pathways. With the abundant information made available by microarray gene expression data (MGED), nonlinear modeling of these interactions is now feasible. Two of the latest advances in nonlinear modeling used sigmoid models to depict transcriptional interaction of a transcription factor (TF) for a target gene, but do not model cooperative or competitive interactions of several TFs for a target.

**Results:**

An S-shape model and an optimization algorithm (GASA) were developed to infer genetic interactions/transcriptional regulation of several genes simultaneously using MGED. GASA consists of a genetic algorithm (GA) and a simulated annealing (SA) algorithm, which is enhanced by a steepest gradient descent algorithm to avoid being trapped in local minimum. Using simulated data with various degrees of noise, we studied how GASA with two model selection criteria and two search spaces performed. Furthermore, GASA was shown to outperform network component analysis, the time series network inference algorithm (TSNI), GA with regular GA (GAGA) and GA with regular SA. Two applications are demonstrated. First, GASA is applied to infer a subnetwork of human T-cell apoptosis. Several of the predicted interactions are supported by the literature. Second, GASA was applied to infer the transcriptional factors of 34 cell cycle regulated targets in *S. cerevisiae*, and GASA performed better than one of the latest advances in nonlinear modeling, GAGA and TSNI. Moreover, GASA is able to predict multiple transcription factors for certain targets, and these results coincide with experiments confirmed data in YEASTRACT.

**Conclusions:**

GASA is shown to infer both genetic interactions and transcriptional regulatory interactions well. In particular, GASA seems able to characterize the nonlinear mechanism of transcriptional regulatory interactions (TIs) in yeast, and may be applied to infer TIs in other organisms. The predicted genetic interactions of a subnetwork of human T-cell apoptosis coincide with existing partial pathways, suggesting the potential of GASA on inferring biochemical pathways.

## Background

Biologists are gradually recognizing that pathways, rather than individual genes, control tumorigenesis [1]. Moreover, altered pathways have recently been reported to be crucial factors for colorectal and breast cancer [2]. The present approach (GASA) was motivated by the inference of genetic interactions, which have potential for inferring pathways in yeast [3,4]. Because genetic networks derived from yeast are likely to be conserved in humans, the prediction of genetic interactions may shed light on the pathways of complex human diseases, such as cancers and type II diabetes. In addition, GASA can also be applied to infer other types of networks, for example transcriptional regulatory networks. With the abundant sets of microarray gene expression data (MGED) now available, inferring genetic interactions has become feasible, and various approaches have been proposed. Most of the approaches may be classified into three classes: graphical models, discrete variable models and continuous variable models. Due to space constraints, here we concentrate our review on continuous variable models that are directly relevant to GASA; see [3] for further reviews of models from other classes.

To approximate the nonlinear relationship of a target (T) and its activator (A) and repressor (R), [5] proposed ordinary differential equations including perturbations from genes of interest; the perturbations provided information of the underlying network topology. The time series network inference algorithm (TSNI) [6] further characterized the perturbations by a few linear external perturbations, and solved the system of equations by using principle component analysis (PCA) and singular value decomposition. On the other hand, network component analysis (NCA) [7] employes partial knowledge of the underlying network and requires no statistical assumption as PCA does [8] and [9] proposed order-two models on interactions of A, R and T. Some latest advances of nonlinear models are as follows. Climescu-Haulica and Quirk proposed a beta-sigmoid function to model the local transcriptional effect on a target in [10]. And Vu and Vohradsky presented a sigmoid model to depict the interaction between a target and its transcriptional factors (TFs) in [11], where order-n polynomials were used to approximate the model. The algorithm was efficient and it predicted more regulators for 40 yeast cell cycle regulated targets than the generalized linear model in [12]. However, the model in [11] is not able to depict cooperative or competitive TFs for a target gene simultaneously. An alternative nonlinear model is the S-system [13,14], which satisfies several cellular processes. However, to model a network of *k *factors regulating a given gene, the proposed sigmoid model requires *n *(*n *+ *k *+ 2) parameters while the S-system requires 2*n *(*n *+ 1). Despite the many merits of the S-system, the large number of parameters required restricts its applications in the area.

To model biological processes occurring simultaneously among a small set of genes such as in genetic interactions, we propose a system of nonlinear equations; for an earlier version of this system see [15]. This model also extends the aforementioned sigmoid models to depict cooperative/competitive interactions among genes. Note that each *tanh *function in this system can include a term, called *factor*, to model genes that regulate other genes but are not regulated by others in the network, and in this sense it extends the linear dynamic factor model in [3].

The transcriptional rate is known to be S-shaped [16]. Let *g*_*i*_(*t*) and Δ*g*_*i *_(*t *+ 1) denote gene *i*'s expression at time t and its expression change at time *t+ *+ 1 respectively, where Δ*g*_*i *_(*t *+ 1) = *g*_*i*_(*t *+ 1) - *g*_*i *_(*t*). This model states that in the network, each gene's expression change is regulated by a weighted sum of certain genes cooperatively at time t only if this weighted (combined) sum surpasses a certain threshold. The proposed nonlinear model depicts the transcriptional rate of gene *i *as follows.(1)

where *ε*_*i*_(*t*) ~*N*(0, ) and  is the variance of gene *i*'s expression levels, which can be estimated from real MGED. For a given gene *i*, |*α*_*i*_| is the range of Δ*g*_*i*_/Δt (the rate of expression change of *g*_*i*_), *β*_*i *_is the location parameter at which the *tanh *function crosses zero, *w*_*ji *_is the regulation of *g*_*j*_(*t*) on Δ*g*_*i*_(*t *+ 1) before being amplified by the *α*_*i *_*tanh*(·) function, *K *is the number of factors *F*_*k*_*'s *in the local network and *K *≤ *n*. Note that [11] can be regarded as a special case of Eq. (1), namely when only one gene inside the *tanh *function regulates *g*_*i*_*'s *transcription rate.

An optimization algorithm, consisting of a genetic algorithm (GA) and a simulated annealing (SA) algorithm, to evolve the optimal genetic network and then estimate the parameters using time course MGED, is developed in the Methods section. This SA is enhanced by a steepest-gradient-descent algorithm to prevent it from being trapped in local minima. A strategy to identify factors in the network is also proposed. The Results section consists of applications of GASA to both simulated data and real time course MGED. First, an S-shape non-linear model (a network) is applied to simulate data with various degrees of noise. Using these generated data, we study how GASA with two model selection criteria and two search spaces performs compared to TSNI, NCA, GA with regular SA (GA-regular SA) and GAGA. Note that GAGA applies a GA algorithm and some model selection criterion to predict networks, but uses another GA to optimize interactions *w*_*ji *_'s in Eq. (1), instead of the enhanced SA in GASA. Second, GASA is applied to real gene expression data sets to infer a partial pathway of human T-cell apoptosis, and the TFs of 34 targets in yeast cell cycle to provide a comparison to TSNI, GAGA and NLDE in [11]. Both predictions are checked against published literature.

## Results and Discussion

When implemented with a factor analysis algorithm, AIC and BIC model selection criteria (MSC) outperform the other four MSC on inferring genetic networks using data simulated from a linear dynamic model [3]. However, the suitable MSC and search space (power law or non-power law) for GASA applied to data from a nonlinear model remain unknown. In this section, we first simulate data from a sigmoid model with various degrees of noise to see how GASA with two MSC and two types of search space perform. The effectiveness of the proposed factor finding strategy and how smoothing circumvents contamination from noise are also studied. Next, GASA is applied to two real datasets to infer a small network of human T-cell apoptosis and the TFs of 34 target genes involved in yeast cell cycle.

### Results on simulated data

Time course data from an 11-gene network including two external factors are simulated. These external factors model TFs or other known proteins, published in the literature, that regulate genes in the network. Two factors *F*_1 _and *F*_2 _were extracted from 51 yeast genes involved in DNA synthesis and repair [17], by applying independent component analysis in MATLAB to these genes' aggregated microarray data of the alpha, cdc15 and cdc28 sets (59 time points in total). The expression profiles of these two factors exhibited sinusoid patterns as shown in Figure 1 of [18]. The coefficients *W*_*ji *_'s and 's in Eq. (2) were determined by trial and error, and the initial values of the 11 genes were generated randomly then tuned manually such that these genes' expression curves also showed sinusoid patterns. For each gene, the initial value and the values of the two factors were plugged into Eq. (2) to recursively generate the rest time points. Simulated gene networks of two factors and 11 genes were generated by the following model.(2)

**Figure 1:**
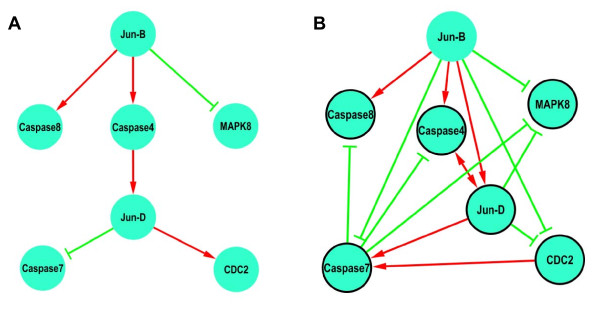
**(A) The network inferred by Rangel *et al*. (2004) with red links denoting enhancing, and green links denoting repression**. (B) The network predicted by GASA using AIC and without the power law restriction in the search space. Black circles around genes denote repressive self-loops.

where *ε*_*i *_~ *N*(0, ),  = *Var*(*g*_*i*_)/*c*, and *c *= 10 or 4 for *i *= 1,..., 11, representing data with signal noise ratio equal to 10 or 4 (denoted by SNR10 and SNR4), respectively. *Var*(*g*_*i*_) was calculated from the sample variance of real microarray data of *g*_*i*_. SNR10 and SNR4 data were generated to mimic data with medium and low quality, respectively, while noise free data were generated without the noise terms *ε*_*i*_(*t*)'s. Note that networks generated by Eq. (2) are sparse which roughly follows the property of *cis*-regulatory networks [19].

Before applying GASA to the datasets, it is necessary to distinguish factors from other genes. We first applied the proposed factor-finding strategy written in Python to the simulated noise free data, and GASA identified the two factors correctly. For data contaminated with noise, mean filters with the kernel size 1 × 3 and 1 × 5 were applied to smooth SRN10 and SRN4 data, respectively. Then, GASA identified the factors correctly; for details see http://www.stat.sinica.edu.tw/~gshieh/GASA/boxplots.pdf.

The performances of GASA with a model selection criterion (MSC) AIC or BIC and with or without the power law restriction (PL or no PL) in the network search space were studied using the three simulated data sets. We also compared GASA to NCA, TSNI, GAGA and GA-regular SA. Both 100% and 50% true connectivity information were inputted into NCA to see how the performances of NCA vary. Table 1, 2 and 3 summarize the comparisons of these algorithms. The true positive rate (TPR), true negative rate (TNR), and modified false positive rate (mFPR) of the predicted top 1 network in terms of AIC (BIC) score, are reported, where TPR (also known as sensitivity) is the percentage of correctly predicted links out of the total number of existing interactions (links) in a simulated network. Similarly, TNR (FPR) is the ratio of correctly predicted non-existing interactions (predicted false positives) over the total true non-existing interactions (negatives) in a simulated network, and mFPR is the ratio of incorrectly predicted interactions to the total predicted ones. There are much more non-existing interactions (117) than existing ones (26) in the true network (Eq. (2)), mFPR can distinguish the performances of the algorithms well, so it is reported in addition to FPR.

**Table 1:** Performances of GASA, TSNI, NCA, GAGA and GA-regular SA applied to one repeat of data simulated from Eq. (2) with no noise.

		**# int**^**a**^	**# pc**^**b**^	**TPR**^**c**^	**TNR**^**d**^	**FPR**^**d**^	**mFPR**^**e**^
GASA	AIC/no power law			0.81	0.99	0.01	0.05
							
	BIC/power law			0.77	0.99	0.01	0.05

GA-regular SA	AIC/no power law			0.69	0.97	0.03	0.18
							
	BIC/power law			0.73	0.97	0.03	0.14

NCA	100% true connectivity			0.62	0.95	0.05	0.27
							
	50% true connectivity			0.24	0.85	0.15	0.75

TSNI	inputting prior knowledge: 26 true links	3	1	0.50	0.89	0.11	0.50
		3	2	0.50	0.89	0.11	0.50
		3	3	0.50	0.89	0.11	0.50

GA-GA	AIC/no power law			0.46	0.79	0.21	0.68
							
	BIC/power law			0.35	0.83	0.17	0.69

^a ^'# int' denotes the number of interpolations.

^b ^'# PC' denotes the number of principal components

^c ^TPR is the ratio of the correctly predicted links to the total number of existing links in a simulated network. Note signs of interactions were not accounted toward TPR and other performance measures.

^d ^TNR (FPR) is the ratio of correctly predicted non-existing links (false positives) over the total true negatives.

^e ^mFPR is the ratio of incorrectly predicted links to the total predicted links.

**Table 2:** Performances of GASA, TSNI, NCA, GAGA and GA-regular SA applied to data simulated from Eq. (2) with medium level of noise, where the averaged results of five repeats are reported

		**# int**^**a**^	**# pc**^**b**^	**TPR**^**c**^	**TNR**^**d**^	**FPR**^**d**^	**mFPR**^**e**^
GASA	AIC/no power law			0.79	0.99	0.01	0.05
							
	BIC/power law			0.79	0.99	0.01	0.05

GA-regular SA	AIC/no power law			0.46	0.97	0.03	0.25
							
	BIC/power law			0.42	0.93	0.07	0.42

NCA	100% true connectivity			0.51	0.92	0.08	0.41
							
	50% true connectivity			0.29	0.86	0.14	0.68

TSNI	Inputting prior knowledge: 26 true links	3	1	0.50	0.89	0.11	0.50
		3	2	0.50	0.89	0.11	0.50
		3	3	0.50	0.89	0.11	0.50

GA-GA	AIC/no power law			0.35	0.78	0.22	0.74
							
	BIC/power law			0.31	0.85	0.15	0.69

^a ^'# int' denotes the number of interpolations.

^b ^'# PC' denotes the number of principal components

^c ^TPR is the ratio of the correctly predicted links to the total existing links in a simulated network. Note signs of interactions were not accounted toward TPR and other performance measure.

^d ^TNR (FPR) is the ratio of correctly predicted non-existing links (false positives) over the total true negatives.

^e ^mFPR is the ratio of incorrectly predicted links to the total predicted links.

**Table 3:** Performances of GASA, TSNI, NCA, GAGA and GA-regular SA applied to data simulated from Eq. (2) with high level of noise, where the averaged results of five repeats are reported

		**# int**^**a**^	**# pc**^**b**^	**TPR**^**c**^	**TNR**^**d**^	**FPR**^**d**^	**mFPR**^**e**^
GASA	AIC/no power law			0.66	0.97	0.03	0.19
	BIC/power law			0.65	0.97	0.03	0.19

GA-regular SA	AIC/no power law			0.5	0.95	0.05	0.30
							
	BIC/power law			0.47	0.96	0.04	0.31

NCA	100% true connectivity			0.58	0.94	0.06	0.31
							
	50% true connectivity			0.25	0.85	0.15	0.73

TSNI	inputting prior knowledge: 26 true links	3	1	0.50	0.89	0.11	0.50
		3	2	0.50	0.89	0.11	0.50
		3	3	0.50	0.89	0.11	0.50

GA-GA	AIC/no power law			0.31	0.83	0.17	0.71
							
	BIC/power law			0.31	0.90	0.10	0.60

^a ^'# int' denotes the number of interpolations.

^b ^'# PC' denotes the number of principal components

^c ^TPR is the percentage of correctly predicted links out of the total number of existing links in a simulated network. Note signs of interactions were not accounted toward TPR and other performance measure.

^d ^TNR (FPR) is the ratio of correctly predicted non-existing links (false positives) over the total true negatives.

^e ^mFPR is the ratio of incorrectly predicted links to the total predicted links.

For each simulated network, we limited the maximum number of incoming links to four for each gene, hence GASA sought through a space of 4^11 ^possibilities. Applying the algorithms to the three simulated datasets, their overall performances in terms of both TPR and mFPR in descending order are GASA, GA-regular SA, NCA (100% true connectivity), TSNI, GAGA and NCA (50% true connectivity). These results were computed without taking the signs of interactions into account. When the signs were also checked, the performance ranking is the same except that GAGA performed better than TSNI; see the additional file 1 for details. Specifically, for noise free data (with one repeat), the TPR, TNR and mFPR of GASA with the four combinations of MSCs and search spaces are similar and equal to 77-81%, 99% and 5%, respectively, whereas those of TSNI are 50%, 89% and 50%, respectively. GAGA has about 30% lower TPR and 60% higher mFPR than GASA using the two combinations BIC/PL and AIC/no PL. From five repeated experiments of SNR10 (SNR4 data), the TPR, TNR and mFPR of GASA with AIC/no PL and GASA with BIC/PL are the same (quite close), and they are both equal to 79%, 99% and 5% (66%, 97% and 19%), respectively; while the performances of TSNI for SNR10 and SNR4 are both equal to 50%, 89% and 50%, respectively. GAGA with AIC/no PL performs similarly to GAGA with BIC/PL, and both have about 35%, 78% and 74% of mTPR, TNR and mFPR. See Table 1, 2 and 3 for detailed performances; the predicted networks and the true one are plotted in additional file 2. The implementation of TSNI and GAGA is summarized in additional file 3. Each simulation of GASA took about 32 h and was conducted by PC cluster (limited to five nodes) with Pentium 3.4 GHz and 4.0 GB RAM; GASA was written in Python 2.1.3 and GNU C. Note that this CPU time can be significantly shortened by using modern multi-core architecture as stated at the end of Application 2.

### Results with real time course microarray data

#### Application 1: A human T-cell apoptosis subnetwork

In [20], two experiments were conducted to characterize the response of a human T cell line (Jurkat) to PMA and ionomycin treatments. The mechanism studied is a key for clonal expansion and controlling long term behavior of T cells such as programmed cell death. Identical experimental protocols were used in the two experiments including more than 88 genes, but only 58 genes with good reproducibility were retained. There were 10 time points, with 34 and 10 replicates for each time point in the first and second experiments, respectively; we called this data set 'human T-cell line data'.

In this 7-gene sub-network, JNK, JUNB (alias name AP-1) and caspase-8 are involved in the apoptosis pathway. As reviewed in [21], apoptosis is a cell suicide mechanism, though which metazoans control cell number in tissues and eliminate individual cells that threaten the animal's survival. The physiological role of apoptosis is extremely important. For instance, unscheduled apoptosis of certain brain neurons leads to diseases such as Alzheimer's disease; and in dividing cells, failure to initiate apoptosis in cells that have serious damages in DNA contributes to cancer. Moreover, JNK (alias name MAPK8) and JunD are involved in the JNK signaling pathways, and JunD inhibits fibroblast proliferation and counteracts transformation by *ras*.

Prior biological information indicated that JUNB was not regulated by other genes in the network, thus JunB was specified as a factor. Next, we applied GASA with AIC/BIC and with/without power law restriction in the search space to the human T-cell line data. The score for GASA with AIC/no PL was the lowest (2526.9), lower than GASA with AIC/PL (2957.3). GASA with AIC/no power law predicted a few interactions that are in the existing pathways; see Figure 1 for details. In particular, JunB activates the apoptotic genes caspase-4 and caspase-8, and represses MAPK8. Moreover, GASA predicted two interactions that are consistent with known protein-protein interactions. Specifically, JunD interacts with MAPK8 which is involved in the JNK signaling pathways in mouse [22]; caspase-4 cleaves and activates its own precursor and caspase-1 precursor (Entrez Gene database at NCBI, http://www.ncbi.nlm.nih.gov/sites/entrez). Caspase-8 activates downstream effectors caspases and commits the cell to apoptosis [21]. Finally, eight testable predictions including three self-regulations were also inferred by GASA. GASA took about 1.4 h using 10-node PC cluster with Intel Xeon 2.0 GHz and 6 GB RAM.

#### Application 2: Inferring the regulators of 34 selected cell cycle regulated targets in yeast

The procedure in [11] (abbreviated as NLDE) is one of the latest advances in nonlinear modeling of transcriptional regulation. Because we could not access the NLDE code, to provide a possible comparison we applied GASA to infer the regulators of 34 yeast cell cycle regulated targets, which were inferred by [11]. Their TFs were collected from YEASTRACT http://www.yeastract.com. The data sets used were cDNA microarray data from three synchronization experiments in [18]; the *Elu *data set was not included because it was synchronized differently. The experiment and control groups were mRNAs extracted from synchronized and non-synchronized yeast cultures, respectively, where the synchronization was conducted by treating yeast cultures with alpha factor arrest and arrests of a temperature-sensitive mutant *cdc15 *and mutant *cdc28*. The red (R) and green (G) fluorescence intensities were measured from the mRNA abundance in the experiment group and control group, respectively. A full description of data preprocessing is available in Additional file 4, and the data sets are available at http://genome-www.stanford.edu/cellcycle/ . We aggregated 18, 24 and 17 time points from these three datasets to 59 time points. This aggregation method was applied in [23] and [3], and it led to meaningful gene networks.

For each cell cycle regulated target, NLDE in [11] predicted one by one whether any of these 184 potential regulators was a true regulator. NLDE is good at screening large numbers of regulators but it can not predict a network of two or more genes simultaneously. To infer TFs for a given target gene, we inputted all 184 TFs simultaneously as potential regulators into one equation in Eq. (1) for GASA to infer. The mTPR* (mFPR) of GASA, GAGA (both using AIC/no PL to select the number of TFs) and TSNI are 65% (45%), 29% (77%) and 8% (92%), respectively, where mTPR* is the ratio of the number of predicted positives over the minimum between the number of validated TFs in YEASTRACT and the total predicted links. TSNI was implemented with no perturbation, three interpolations and three principle components, which were obtained by the curve maximizing the area of TNR versus TPR as [6]; the results of one and two principle components are also reported. Note that YEASTRACT consists of both documented regulators (experimentally confirmed to date) and potential regulators (predicted by matching TF promoter and TFBS). The confirmed TFs therein were used (Yeastract_TFs_34_targets.pdf at http://www.stat.sinica.edu.tw/~gshieh/GASA, but they are far from complete, therefore the mFPRs computed here may be higher than they should be. We could not access the executive code of NLDE; however, summarizing from Table 2 of [11], the mTPR* and mFPR of NLDE are about 2% and 98%, respectively. These prediction accuracies are presented to serve as contrasts, not as direct comparisons. See Application2.pdf at http://www.stat.sinica.edu.tw/~gshieh/GASA for details of the results. On average GASA took about 88 min to predict TFs for each target using one node from a mini-cluster (six Intel i7-950 with 3.06 GHz per node sharing 24 GB RAM, and accelerated by 480 GPU per node sharing 2 GB RAM.

## Discussion

The prediction results of simulated data show that GASA can infer small networks more accurately than TSNI, NCA, GAGA and GA-regular SA. The results of inferring TFs for the 34 cell-cycle regulated targets using real gene expression data in Application 2 suggest that GASA characterizes the nonlinear transcriptional regulation better than NLDE, GAGA and TSNI. Furthermore, the results of simulation study and Application 2 indicate that SGD-enhanced SA helps infer the transcriptional interactions (*w*_*ji *_'s in Eq. (1)), and results in accurate networks.

Due to intensive computation of systems of nonlinear equations, initial networks inferred by GASA are limited to 10+ genes and factors. Nevertheless, these small networks can be extended to large ones using the following two schemes. First, a large network can be partitioned into a few small networks using biological information, then GASA can be applied to infer each small network. For instance, to infer transcriptional compensation interactions from synthetic sick or lethal (SSL) gene pairs, one can partition a network of 200+ genes in [17] into a few small networks via SSL pairs. Moreover, for inferring transcriptional regulatory interactions, a large network can be divided into small networks centering on several TFs or target genes [4]. Second, a few inferred small networks can be integrated into a large network using a merging scheme. This scheme can be applied iteratively to result in a fairly large network. Below we illustrate how two small gene networks can be merged to a large one using the network in Eq. (2).

Given two small networks, e.g. Γ_1 _consisting of the first four equations of Eq. (2) and Γ_2 _the last seven equations. Specifically, Γ_1 _= {f_1_, f_2_, g_1_, g_2_, g_3_, g_4_} and Γ_2 _= {f_1_, f_2_, g_5_, g_6_, g_7_, g_8_, g_9_, g_10_, g_11_, g_12_, g_13_}. We first calculate the SSE/Var of each gene in the network Γ_1_. Next, genes of Γ_2 _are added one by one into Γ_1_, then SA is applied to estimate parameters of each potential link and calculate its SSE/Var value. The potential link with the highest score of SSE/Var of g_1 _and its associated gene is added to Γ_1 _(which forms the merged network Γ'_1_). Then, calculate the fitness score (AIC or BIC) of Γ'_1 _to check network complexity; if Γ'_1 _is better than Γ_1_, then Γ_1 _is updated to Γ'_1_. These procedures are repeated for the rest of genes in Γ_1_. Similarly, the same procedures can be repeated to integrate the genes of Γ_1 _and the associated links to Γ_2_. We have conducted an experiment on these small networks Γ_1 _and Γ_2 _using data simulated from Eq. (2) with no noise and SNR4. The TPRs and TNRs of the merged networks are compatible to those inferred from one whole network, but the FPR of the merged network is a little worse than that inferred from one network; the merging scheme and the results are in Additional file 5.

## Conclusions

GASA extended one of the latest advances in nonlinear modeling of transcriptional interactions [11] to: (1) infer cooperative/competitive interactions, and (2) infer genetic networks/transcriptional networks of a few genes simultaneously. In particular, when inferring TFs for 34 yeast cell cycle regulated targets in yeast, GASA has an averaged mTPR about 65% and mFPR 46%, which performs better than NLDE, GAGA and a linear model based algorithm (TSNI). For inferring transcriptional interactions of higher organisms such as 2000+ TFs in human, we can apply biological knowledge, e.g. using differentially expressed genes at one or more time points or/and incorporating experimental conditions, to narrow down the TFs to a few hundreds genes of interest [24], then apply GASA as demonstrated in Application 2. Moreover, several predicted genetic interactions of GASA in the human T-cell subnetwork are consistent with protein-protein interactions in human/mouse JNK signaling/T-cell apoptosis pathways, which suggests that GASA might be applied to predict biochemical pathways. In the simulated networks, GASA identified factors correctly and outperformed NCA, TSNI, GAGA and GA-regular SA. Although the scale of the simultaneous networks, e.g. Eq. (1), inferred by GASA is limited to 10+ genes due to intensive computation, the inferred networks can be expanded either by integration of several small inferred networks into a large one or partitioning a large network into several small ones and then applying GASA as stated in the Discussion section. Recently, other types of genomics data such as ChIP-chip have become available. Incorporating ChIP-chip data to identify a small set of plausible regulators for a target then applying GASA to infer the regulatory/genetic relationship seems promising [25]. This would also allow GASA to infer large networks in addition to improving the prediction accuracy. We leave this avenue for exploration in future research.

## Methods

### Applying a mean filter to smooth microarray data

To dampen noise in MGED, a mean filter [26] in the discrete signal processing area was applied. A mean filter with kernel size *r *× *c *can be viewed as a window of size *r *× *c *centered at an original datum, and replacing the datum (pixel) with the average of all pixels in the window. See mean_filter.pdf http://www.stat.sinica.edu.tw/~gshieh/GASA/mean_filter.pdf of Supplementary data or Figure 2 of [4] for the effects of mean filters on MGED. In the simulation study of the Results section, mean filters were applied, then the factor-identifying strategy (stated below) found the factors correctly when simulated data have noise.

### A strategy to identify factors in a network

Factors are defined as special genes if they regulate (have only outgoing links to) other genes within a closed network of interest. They may have incoming links from genes outside the closed network but not from within. With this definition in mind, if GASA treats the factors as other regulated genes and tries to find their incoming links from genes within the closed network, then this will result in a relatively large *SSE/Var*, which indicates a lack of fit.

Therefore, assuming there is no factor in the closed network, we calculate the smallest *SSE/Var *of each gene as follows. First, we set the maximum number of incoming links of each gene to *l*_max _and enumerate all possible combinations. The smallest *SSE/Var *of a given gene can be obtained by applying the SGD algorithm to enumerate each combination. Then the components that have extremely large values of *SSE/Var *(namely the outliers in the boxplot of all *SSE/Var *values) can be identified as factors; see http://www.stat.sinica.edu.tw/~gshieh/GASA/boxplots.pdf for examples. Moreover, recently there have been several ChIP-chip data sets published. These data can also provide *a priori *information on potential TFs for a given target gene. This data could be used to identify factors before implementing GASA. Integrating ChIP-chip into GASA is a promising future work.

### The search space of networks

Finding the optimal genetic network with *n *genes involves the estimation of *n*(*n *+ *k *+ 2) parameters in the model of Eq. (1). Ideally, these unknown parameters may be determined using a global optimization technique provided that there is sufficient data. However, it is intractable in practice when *n *is large since this is NP-complete [27]. The computation time of the task increases exponentially with *n*.

To circumvent this intractable problem, the problem of finding the optimal fully-connected network in a search space of dimension *n*(*n+k*) is reformulated to finding the best network among some optimal partially-connected networks. This is feasible because in general the matrix of connectivity *W *in Eq. (1) is sparse [19]. There are two sets of unknown parameters: the number of partially-connected networks (structures) and 2*n + L*(*π*) parameters, where *L*(*π*) denotes the number of links in a given partially-connected network *π*. Specifically, GASA searches through the space of partially-connected gene networks via a GA and for each fixed structure *π*, we estimate the associated 2*n + L*(*π*) parameters by an SA algorithm. The optimal gene network is the one that minimizes the cost function. More details of GASA are given in Subsections Parts 1 and 2 of GASA.

Reducing a high dimensional problem to a set of low dimensional problems will substantially save computation time. To reduce the search space of *π*, we set a limit on the number of incoming links of each gene by *l*_max_. Therefore, when determining the combination of incoming links for each gene, the size of search space reduces from 2^*n *+ *k *^to . Moreover, when the number of genes is large, many of their cellular networks follow a power law [28], which states that the probability of *k *interactions (incoming links for each gene) decays according to a negative power of *k*, namely *P*(*K *= *k*) ~ *k*^-*γ*^, where 2<*γ*<3. Therefore, we may further restrict the search space to follow the power law.

### GASA Part 1-GA

Given the factors, we implement a GA as follows. First, we encode a network structure by a *n*(*n *+ *k*)-bit array or a *n *by *n *+ *k *bit-matrix, where a 1 at position (*i, j*) represents a link from *g*_*j *_to *g*_*i*_, while a 0 denotes no links. Let the maximum number of links be *l*_max_, then the number of bits required to encode a network structure into a chromosome is reduced to *n *× *l*_max_. In the GA part of GASA, a network structure uses *l*_max _bits to specify the in-degree of each gene, which is the number of 1's in the *l*_max _bits. Therefore, given a network structure or a chromosome, we enumerate all possible sets of incoming links for each gene, and retain the set with the smallest value of *SSE*(*g*_*i*_), where(3)

and (*t*) the predicted expression level of gene *i *at time *t *with the estimated parameter; the *SSE *can be obtained after SA (in the subsection GASA Part 2) estimates the parameters. The self-link for each gene is also considered but it is not counted toward the number of incoming links.

Starting with a population of *N *chromosomes, these chromosomes in a simple GA evolve mimicking natural mechanisms such as selection, crossover and mutation. While selection may be considered as an evolution operator, crossover and mutation serve as genetic operators. In each generation, the evolution process starts with *N *chromosomes, called parent chromosomes. A fitness score, AIC or BIC score, is first computed for each parent chromosome, where

and *Var*(*g*_*i*_) is the sample variance of *g*_*i *_across time.

Next, new chromosomes are generated by the genetic operators. The operator crossover exchanges the same segments of two parent chromosomes to create two new child chromosomes. One-point crossover is implemented in GASA, and crossover is not applied to the end points of each chromosome to avoid inefficient perturbation. These two parent chromosomes are chosen randomly without replacement. On the other hand, the operation mutation perturbs the steady state of each chromosome by randomly toggling very few bits to evolve into new child chromosomes. The probability of being selected for mutation is inversely proportional to the fitness score. Namely, the lower the fitness score of a chromosome, the better chance it will be selected for mutation to reach a higher fitness value.

After the fitness score of each new child chromosome is computed, as in any conventional GA, a new generation of *N *chromosomes is selected from the pool of parent and child chromosomes. The selection rules vary from one algorithm to another, for instance, fitness-proportional selection, rank-based selection, elitist selection, and others [29]. Our system selects *N/*2 chromosomes with the highest fitness values from the pool of parent chromosomes and from child chromosomes, respectively. As such, GA evolves through generations until the best fitness scores of generations converge.

### GASA Part 2: Stochastic Gradient Descent-enhanced SA

From the model in Eq. (1), it is clear that (*t+ *1) depends on *g*_*i*_(*t*) 's but not on (*t*) 's. Thus minimizing *SSE*(*g*_*i*_) is independent from minimizing *SSE*(*g*_*j*_) when *i *≠ *j*. For each structure *π *generated by GA, the parameters will be estimated by an SA gene-wise which is a stochastic search method that incorporates randomness in the search process. SA simulates the annealing process that cools a molten substance into a crystalline solid. Since the quality and stability of a crystalline solid depends on the cooling process, annealing may be regarded as an optimization process searching through the space of the unknown parameters. In an SA, the temperature *T *decreases from an initial high temperature gradually. At a given temperature *T*, the probability that the system stays in a particular state *s *(*p*(*s*, *T*)) depends on the free energy *E*(*s*), and it follows the Boltzmann distribution [30]

where  and *k *is the Boltzmann constant.

The main idea of an SA algorithm is to search for the minimum energy at each temperature *T*, and this is usually achieved by the Metropolis-Hastings (MH) algorithm [31,32]. In the MH algorithm, a move from state *s*_1 _to state *s*_2 _follows the rules that (i) if *E*(*s*_2_) <*E*(*s*_1_), then state *s*_2 _is accepted, otherwise (ii) the move is rejected with probability 1-*exp*{[*E*(*s*_2_) - *E*(*s*_1_)]/*kT*}.

The rule (ii) distinguishes the SA algorithm from other gradient descent approaches. By allowing the system to move into a state with a higher energy, this SA algorithm inherently can escape from the local minimums. Furthermore, it is shown that the SA achieves the global minimum given a sufficient number of movements. Let *π*_*i *_denote the incoming links of *g*_*i*_, *L*(*π*_*i*_) the number of incoming links of *g*_*i*_, and  the parameter vector to be optimized by the SA algorithm. The cost function to be minimized is defined as

where SSE(*g*_*i*_) is defined in Eq. (3), and *Θ*(*π*_*i*_) denotes a plausible state in the search space. That is, *Θ*(*π*_*i*_) is a state vector and *Θ*(*π*_*i*_) = {*α*_*i*_, *w*_*ji*_, *w'*_*ki*_, *β *_*i*_}, where *j *= 1,..., *n *and *k *= 1,...,*K*. If there are no links from gene *j *to gene *i *or from factor *k *to gene *i *in *π*_*i*_, the corresponding *w*_*ji *_or *w'*_*ki *_is fixed at 0 during the optimization process.

To find the optimal set of *Θ*(*π*_*i*_), one naive approach is to generate new state vector *Θ*(*π*_*i*_) randomly and apply the MH algorithm to determine the movement from one state to another. Although this approach may converge to a global minimum in theory, the computational time may be too intensive to carry out. Alternatively, we propose a stochastic gradient descent (SGD) algorithm to accelerate the convergence process as follows.

1. Initialize *Θ*(*π*_*i*_)

2. Initialize control parameters: *t *← *T*_*Max *_*p *← *1*

3. Compute

4. Compute *Θ*^*new*^(*π *_*i*_):

Generate a uniformly distributed random number in the interval [0, 1], namely *r *~ *U(0, 1)*.

*P*_*Gradient *_← *0.2+0.3t/T*_*Max*_

a. If *r *>*P*_*Gradient*_:

*Θ*^*new*^(*π*_*i*_) ← *Θ *(*π*_*i*_) - *λ*∇*E*(*Θ*(*π*_*i*_)), where *λ *is a damping constant.

b. Else:

5. Update *Θ*(*π *_*i*_):

If E(*Θ*(*π*_*i*_)) > E(*Θ*^*new*^(*π*_*i*_)):

*Θ*(*π*_*i*_) ← *Θ*^*new*^(*π*_*i*_)

Else:

*P*_*MH *_← *exp((E(Θ(π_*i*_)) *- *E(Θ^*new*^(π_*i*_)))/Kt)*, where *K *is a constant.

Generate a random number *r *from *U(0,1)*.

If r <*P*_*MH*_:

*Θ*(*π*_*i*_) ← *Θ*^*new*^(*π*_*i*_)

6. Check for convergence at temperature *t*:

If the stop condition at temperature *t *is met:

*E*_*t*_*(Θ(π*_*i*_)) ← *E(Θ(π*_*i*_)), where *E*_*t*_*(Θ(π*_*i*_*)) *is the energy at temperature *t*.

GOTO step 7

Else:

GOTO step 3

7. Check for convergence throughout consecutive *t*'s

If *t + N*_*C *_<*T*_*Max *_and *|E*_*t *+ *k *+ 1_*(Θ(π*_*i*_)) - *E*_*t *+ *k*_*(Θ(π*_*i*_*))| *<*ε *for *k = 1, 2,..., N*_*C*_, where *ε *and *N*_*C *_are constants:

*Ep(Θ(π*_*i*_)) ← *E*_*t*_(*Θ(π*_*i*_*))*

*Θ*_*p*_*(π *_*i*_) ← *Θ(π*_*i*_)

*p *← *p *+ *1*

Else:

*t *← *t *- *1*

 GOTO step 3

8. Check the effect of perturbing network parameters

If *p *>*N*_*F*_+ *1 *and *E(Θ*_*k*_*(π*_*i*_*)) > E(Θ*_*p*-1-*F*_*(π *_*i*_*)) *for *k = p-F, p-F+1*,..., *p*-*1*, where *N*_*F *_is a constant:

SGD completed, outputting *Θ*_*best*_(*π*_*i*_)

Else:

*Θ(π*_*i*_) ← *{r*_*k*_*θ*_*k*_|*θ*_*k *_in *Θ*_*best*_*(π*_*i*_*)}*, where *r*_*k *_~ *U(0.75, 1.25)*

*t *← *T*_*Max*_

 GOTO step 3

In the SGD algorithm, step 3 is to derive the direction of the steepest gradient descent and step 4a is to update the parameter vector along that direction, which in general leads to a faster convergence. However, the direction of the steepest gradient descent may be trapped in a local minimum. To remedy this drawback, in step 4b we introduce randomness into the converging direction so that the global minimum may be achieved by the second rule of the MH algorithm in step 5. Step 7 checks the absolute difference of energy between pairs of two adjacent temperature points. If the difference is smaller than a constant *ε *for *N*_*C *_successive pairs, then the optimization process converges. To avoid converging to a local minimum, the algorithm repeatedly perturbs the best *Θ(π*_*i*_*) *obtained, and iterates the optimization process until no improvements on *E(Θ(π*_*i*_*)) *can be made for *N*_*F *_times.

**A preliminary version of this article was accepted by IEEE, BMEI conference proceedings, 2009**.

## Authors' contributions

CMC and CL devised the method. CL, CLC and CC implemented the method. GSS conceived the research. CMC and GSS supervised the methodology and implementation, and wrote the manuscript. All of the authors read and approved the final manuscript.

## Supplementary Material

Additional file 1 **Simulation_results_signs_checked.pdf**. Performances of GASA, TSNI, NCA, GAGA and GA-regular SA applied to data simulated from Eq. (2) with no, low to high level of noise, where signs of interactions were counted. Click here for file

Additional file 2 **Fig_predicted_network.pdf**. Predicted networks and the true one presented in Table 1, 2 and 3 from one experiment. Click here for file

Additional file 3 **Implementation_TSNI_GAGA.pdf**. Detailed procedures of the implementations of TSNI and GAGA. Click here for file

Additional file 4 **Data-preprocessing.pdf**. A detailed description of data pre-processing of Application 2. Click here for file

Additional file 5 **Merging.pdf**. Detailed procedures of the network merging scheme and the preliminary results of an experiment. Click here for file
