# Supplementary material for: Inferring genetic interactions via a nonlinear model and an optimization algorithm
Source: BMC Syst Biol. 2010 Feb 26;4:16. doi: 10.1186/1752-0509-4-16 (PMC2848194; doi:10.1186/1752-0509-4-16)
Supplement: Additional file 1 — Simulation_results_signs_checked.pdf. Performances of GASA, TSNI, NCA, GAGA and GA-regular SA applied to data simulated from Eq. (2) with no, low to high level of noise, where signs of interactions were counted. [file 1752-0509-4-16-S1.pdf]

Tables

Table 1 - Performances of GASA, TSNI, NCA, GAGA and GA-regular SA applied to one repeat of data simulated from Eq. (2) with no noise, where signs of W_{ji}'s were counted toward TPR, etc. **TPR is the percentage of correctly predicted links out of the total number of existing links in a simulated network. Similarly, TNR (FPR) is the ratio of correctly predicted non-existing links (false positives) over the total true negatives, and mFPR is the ratio of incorrectly predicted links to the total predicted links.**

		# int[1]	# pc[2]	TPR	TNR	FPR	mFPR
GASA	AIC/no power law			0.77	0.99	0.02	0.09
	BIC/power law			0.77	0.99	0.02	0.09
GA-regular SA	AIC/no power law			0.62	0.97	0.03	0.2
	BIC/power law			0.6	0.97	0.03	0.2
NCA	100% true connectivity			0.41	0.93	0.07	0.45
	50% true connectivity			0.19	0.85	0.15	0.77
GA-GA	AIC			0.42	0.79	0.21	0.69
	BIC			0.31	0.83	0.17	0.71
TSNI	inputting prior knowledge: 26 true links	3	1	0.19	0.82	0.18	0.88
		3	2	0.19	0.82	0.18	0.88
		3	3	0.24	0.83	0.17	0.85

[1] '# int' represents the number of interpolations.

[2] '# PC' represents the number of principal components

Table 2 - Performances of GASA, TSNI, NCA, GAGA and GA-regular SA applied to data simulated from Eq. (2) with medium level of noise, where signs of W_{ji}'s were counted toward TPR, etc. These performances were the averaged results of five repeats. TPR is the percentage of correctly predicted links out of the total number of existing links in a simulated network. Similarly, TNR (FPR) is the ratio of correctly predicted non-existing links (false positives) over the total true negatives, and mFPR is the ratio of incorrectly predicted links to the total predicted links.

		# int[1]	# pc[2]	TPR	TNR	FPR	mFPR
GASA	AIC/no power law			0.77	0.99	0.02	0.09
	BIC/power law			0.77	0.99	0.02	0.09
GA-regular SA	AIC/no power law			0.41	0.97	0.03	0.27
	BIC/power law			0.35	0.96	0.04	0.36
NCA	100% true connectivity			0.38	0.92	0.08	0.52
	50% true connectivity			0.17	0.85	0.15	0.8
GA-GA	AIC			0.31	0.78	0.22	0.76
	BIC			0.27	0.85	0.15	0.72
TSNI	inputting prior knowledge: 26 true links	3	1	0.19	0.82	0.18	0.88
		3	2	0.24	0.83	0.17	0.85
		3	3	0.24	0.83	0.17	0.85

[1] '# int' represents the number of interpolations.

[2] '# PC' represents the number of principal components

Table 3 - Performances of GASA, TSNI, NCA, GAGA and GA-regular SA applied to data simulated from Eq. (2) with high level of noise, where signs of W_{ji}'s were counted toward TPR, etc. These performances were the averaged results of five repeats. TPR is the percentage of correctly predicted links out of the total number of existing links in a simulated network. Similarly, TNR (FPR) is the ratio of correctly predicted non-existing links (false positives) over the total true negatives, and mFPR is the ratio of incorrectly predicted links to the total predicted links.

		# int[1]	# pc[2]	TPR	TNR	FPR	mFPR
GASA	AIC/no power law			0.62	0.97	0.05	0.27
	BIC/power law			0.58	0.97	0.06	0.32
GA-regular SA	AIC/no power law			0.37	0.95	0.05	0.39
	BIC/power law			0.35	0.96	0.04	0.36
NCA	100% true connectivity			0.33	0.9	0.1	0.61
	50% true connectivity			0.14	0.84	0.16	0.84
GA-GA	AIC			0.27	0.83	0.17	0.74
	BIC			0.27	0.90	0.10	0.63
TSNI	inputting prior knowledge: 26 true links	3	1	0.19	0.82	0.18	0.88
		3	2	0.24	0.83	0.17	0.85
		3	3	0.24	0.83	0.17	0.85

[1] '# int' represents the number of interpolations.

[2] '# PC' represents the number of principal components
