# Supplementary material for: Inferring genetic interactions via a nonlinear model and an optimization algorithm
Source: BMC Syst Biol. 2010 Feb 26;4:16. doi: 10.1186/1752-0509-4-16 (PMC2848194; doi:10.1186/1752-0509-4-16)

A predicted network from simulated data set 1, plotted against the true network

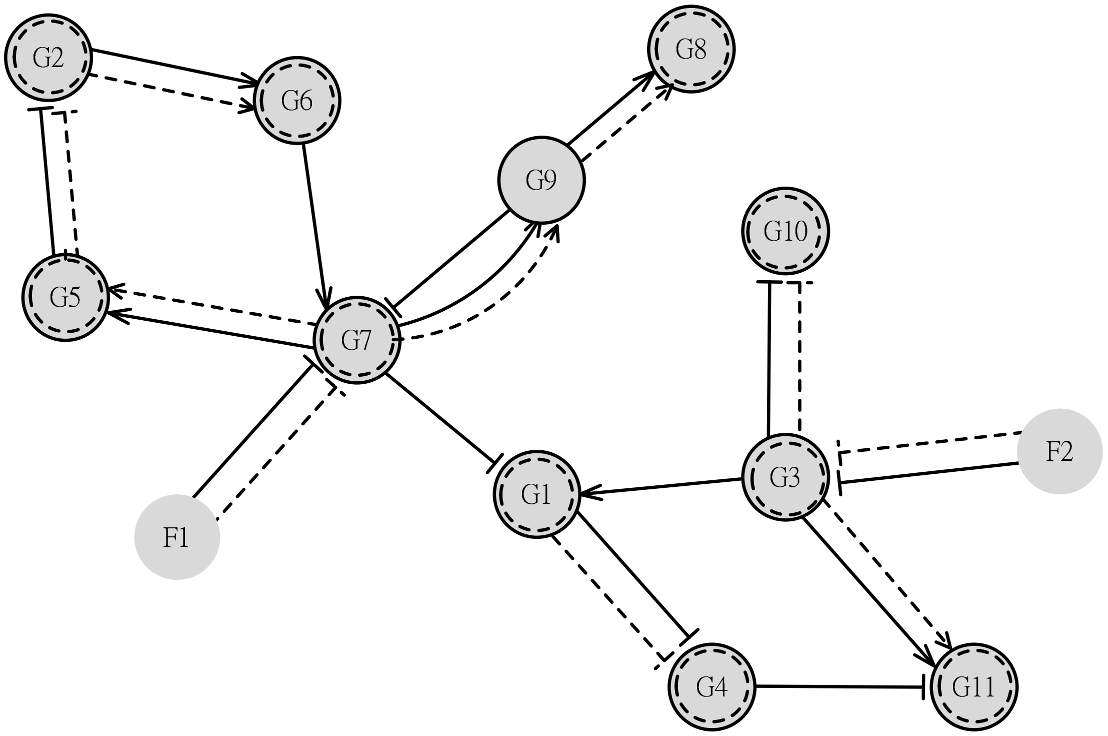

Gi → Gj: Gi having positive regulation on Gj

Gi ─┤ Gj: Gi having positive regulation on Gj:

Gi ─ looping along itself: Gi having positive or negative regulation on itself

─ : True links

- - -: Predicted links

A predicted network from simulated data set 2, plotted against the true network

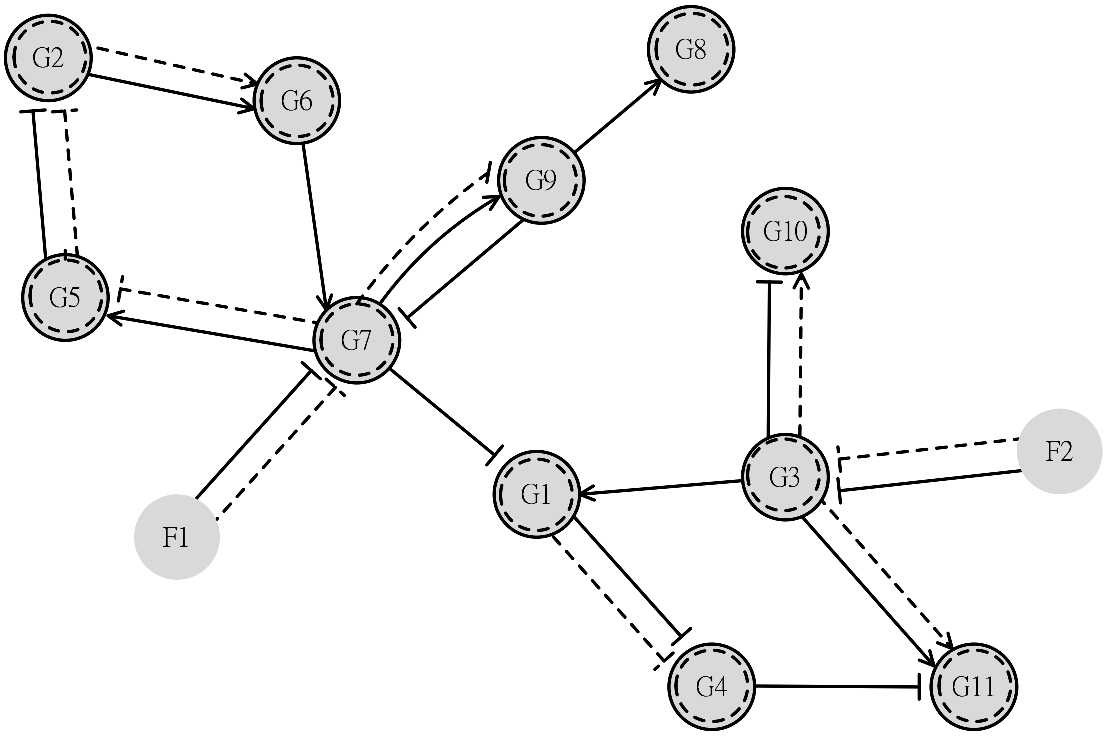

A predicted network from simulated data set 2, plotted against the true network

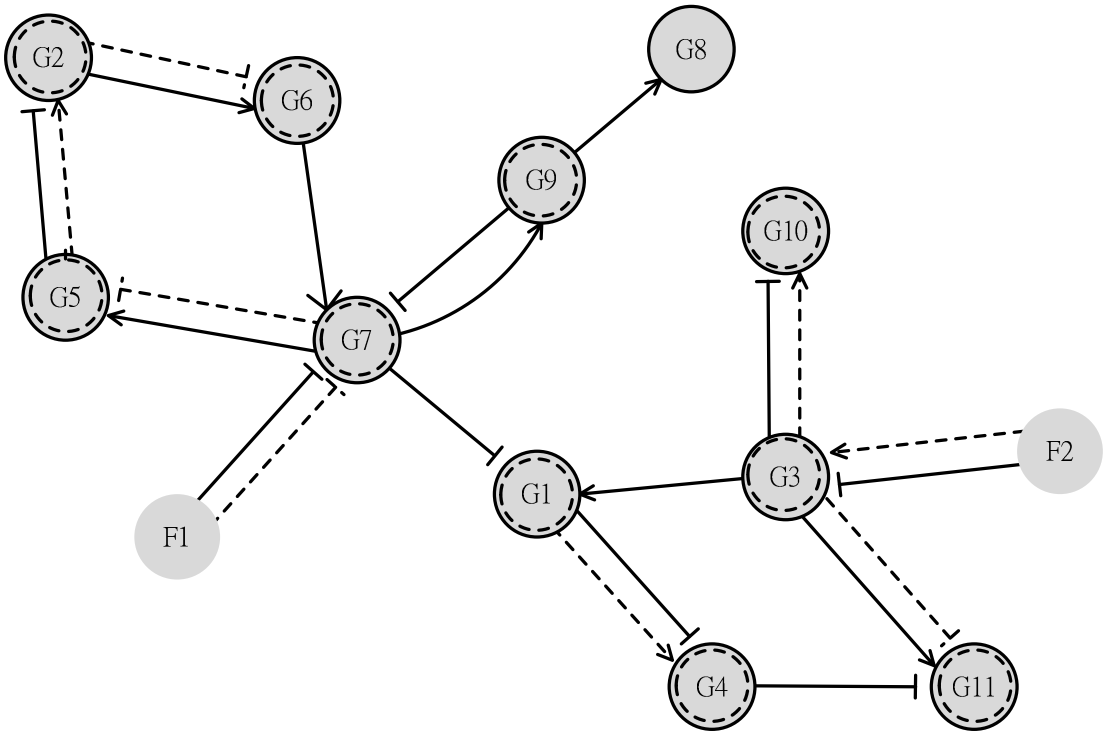

Supplement: Additional file 2 — Fig_predicted_network.pdf. Predicted networks and the true one presented in Table 1, 2 and 3 from one experiment. [file 1752-0509-4-16-S2.pdf]
