# Supplementary material for: Inferring genetic interactions via a nonlinear model and an optimization algorithm
Source: BMC Syst Biol. 2010 Feb 26;4:16. doi: 10.1186/1752-0509-4-16 (PMC2848194; doi:10.1186/1752-0509-4-16)
Supplement: Additional file 3 — Implementation_TSNI_GAGA.pdf. Detailed procedures of the implementations of TSNI and GAGA. [file 1752-0509-4-16-S3.pdf]

Implementation of TSNI:

1. Download TSNI algorithm from http://dibernardo.tigem.it/wiki/index.php/Time_Series_Network_Identification_TSNI
2. Interpolate the time-series microarray gene expression data (MGED) by cubic splines to $\#int \times M$ time points, where M is the number of time points in original time-series MGED.
3. Inputting the number of principal components (#PC) and the interpolated time-series MGED to the MATLAB code of TSNI. Since we do not have the perturbation data, we set all perturbations to zero.
4. All elements in the connectivity matrix inferred by TSNI are sorted by its absolute values.
5. We set the smallest h elements in the inferred connectivity matrix to zero. To plot the r_z vs r_{nz} plot, we varied h from 0 to the number of elements in the inferred connectivity matrix, and evaluate the results by the definitions of r_z and r_{nz}.

$$r_z = \frac{\text{\# of correctly identified zero coefficients in the recovered connectivity matrix}}{\text{\# of zero elements in true network}}$$

$$r_{nz} = \frac{\text{\# of non-zero coefficients with correct signs in the inferred connectivity matrix}}{\text{\# of non-zero elements in true network}}$$

6. The setting (#int, #PC) that yields the maximum area in the r_z vs r_{nz} plot to evaluate the performance of TSNI => (#Interpolation, #PC) =(3,3) for simulation in GASA article.

Area of r_z vs r_{nz} using different setting of (#int, #PC).

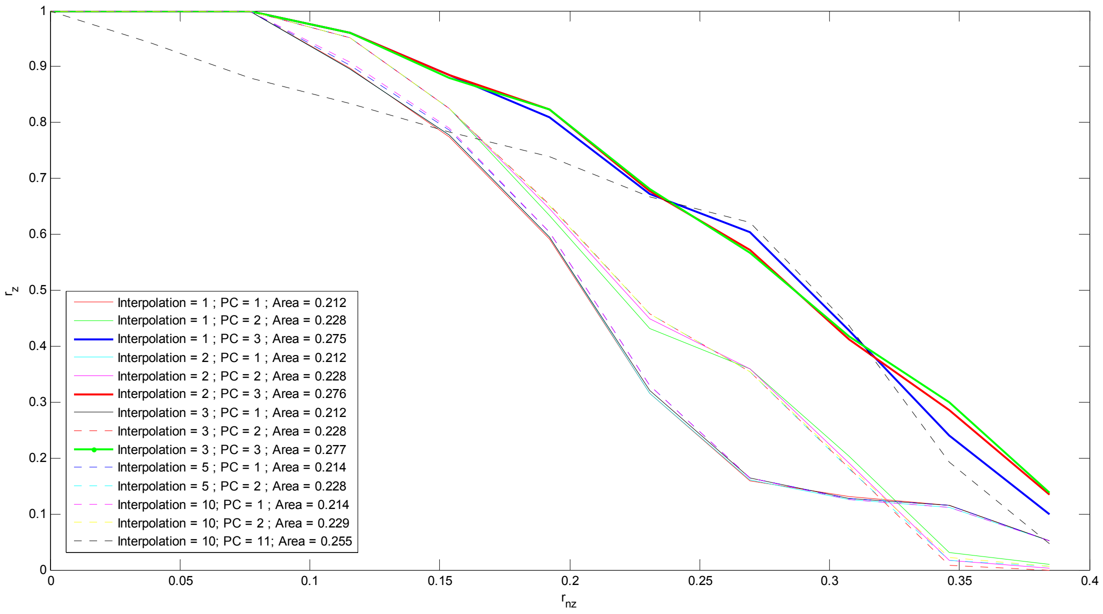

Interpolation = 1 ; PC = 1 ; Area = 0.212
Interpolation = 1 ; PC = 2 ; Area = 0.228
Interpolation = 1 ; PC = 3 ; Area = 0.275
Interpolation = 2 ; PC = 1 ; Area = 0.212
Interpolation = 2 ; PC = 2 ; Area = 0.228
Interpolation = 2 ; PC = 3 ; Area = 0.276
Interpolation = 3 ; PC = 1 ; Area = 0.212
Interpolation = 3 ; PC = 2 ; Area = 0.228
Interpolation = 3 ; PC = 3 ; Area = 0.277
Interpolation = 5 ; PC = 1 ; Area = 0.214
Interpolation = 5 ; PC = 2 ; Area = 0.228
Interpolation = 10; PC = 1 ; Area = 0.214
Interpolation = 10; PC = 2 ; Area = 0.229
Interpolation = 10; PC = 11; Area = 0.255
r_z
r_{nz}

Implementation of GAGA:

1. Use the genetic algorithm presented in the manuscript to find the optimal structure of the regulatory network (according to the fitness function AIC or BIC).
    - All settings are identical to the genetic algorithm presented in the manuscript.
2. Use ordinary genetic algorithm to optimize the weights of the links of the potential networks inferred by **Step 1**.
    - Maximum generation = 10000 generations.
    - Early stop criterion: no better solution for 1000 generations.
    - Chromosome configuration:
        i. The values of the weights are formatted in a fix-point representation
        ii. Length of the chromosome for each weight = 23 bits
            - Sign bit: 1 bit
            - Integer: 6 bits
            - Decimal fraction: 16 bits
            - Range of possible values: +64.99~ -64.99
            - Resolution of the decimal fraction is 0.0000153
    - After all weights are optimized, we evaluate the results by fitness functions (AIC and BIC) defined in the manuscript.
    - Return the weights of the inferred network structures and its fitness to **Step 1** to find a better network structure.

Results of GAGA in the simulation study:

		fitness	TPR	TNR	FPR	mFPR
with sign	no noise	AIC	0.42	0.79	0.21	0.69
		BIC	0.31	0.83	0.17	0.71
	SNR 10	AIC	0.31	0.78	0.22	0.76
		BIC	0.27	0.85	0.15	0.72
	SNR 4	AIC	0.27	0.83	0.17	0.74
		BIC	0.27	0.90	0.10	0.63
no sign	no noise	AIC	0.46	0.79	0.21	0.68
		BIC	0.35	0.83	0.17	0.69
	SNR 10	AIC	0.35	0.78	0.22	0.74
		BIC	0.31	0.85	0.15	0.69
	SNR 4	AIC	0.31	0.83	0.17	0.71
		BIC	0.31	0.90	0.10	0.60

TPR = # of TP / # of known links

TNR = # of TN / # of known negative links

FPR = # of FP / # of known negative links

mFPR = # of FP / # of predicted links

‘with sign’ means non-zero coefficients with correct sign are treated as true-positive; otherwise, they are classified as false-positive.

‘no sign’ means signs of non-zero coefficients are ignored during the evaluation.
