# Supplementary material for: Inferring genetic interactions via a nonlinear model and an optimization algorithm
Source: BMC Syst Biol. 2010 Feb 26;4:16. doi: 10.1186/1752-0509-4-16 (PMC2848194; doi:10.1186/1752-0509-4-16)
Supplement: Additional file 4 — Data-preprocessing.pdf. A detailed description of data pre-processing of Application 2. [file 1752-0509-4-16-S4.pdf]

**Additional file: Data-preprocessing**

There are three sets of data synchronized by treating alpha pheromone (the alpha data set) and temperature sensitive mutation (cdc15 and cdc28) in Spellman et al. (1998). Using these three sets of gene expression data, there are 59 time points in total for each gene. However, the last two time points of cdc15 were deleted to make the phases in a cell cycle in coherent order. In each set of microarray gene expression data, we deleted genes which have missing rate higher than 30% since imputation of these heavily missing data may be problematic. These data used in the Application section are log ratios of red to green channel intensities of cDNA microarray, where the red (green) channel intensities are gene expression levels of synchronized (non-synchronized) yeast. Gene expression from synchronized yeast cells are proportional to mRNA expression levels of *normal* yeast cells while those non-synchronized ones serve as background signals.
Let $R_i(t)$ and $G_i(t)$ be the red and green intensity of gene *i*. These data were normalized by Spellman et al. (1998) such that for a fixed *i*, $\sum_{t=1}^{T} \log_2 \left[ R_i(t) / G_i(t) \right] = 0$, namely $\sum_{t=1}^{T} \log_2 R_i(t) = \sum_{t=1}^{T} \log_2 G_i(t)$. For details, we refer to yeast cell cycle project of the Stanford Genome database (http://genome-www.stanford.edu).

**Data imputation**

To impute those missing data, we applied the k-means clustering to 6,056 genes, and treating each missing cell as the centroid of each cluster. Next, we grouped genes which had correlation, computed from other non-missing data, with the centroid across time ($r_T$) greater than *0.7* into one cluster, where $g_i(t) = \log_2 \left[ R_i(t) / G_i(t) \right]$ and $r_T = \sum_{t=1}^{18} (g_i(t) - \bar{g}_i)(g_j(t) - \bar{g}_j) \Big/ \left[ \sum_{t=1}^{18} (g_i(t) - \bar{g}_i)^2 \sum_{t=1}^{18} (g_j(t) - \bar{g}_j)^2 \right]^{1/2}$. For a fixed time *t*, each missing value of the centroid was imputed with the average of top-10 or fewer (if fewer existing in the cluster) correlated genes.
