# Supplementary material for: Inferring genetic interactions via a nonlinear model and an optimization algorithm
Source: BMC Syst Biol. 2010 Feb 26;4:16. doi: 10.1186/1752-0509-4-16 (PMC2848194; doi:10.1186/1752-0509-4-16)
Supplement: Additional file 5 — Merging.pdf. Detailed procedures of the network merging scheme and the preliminary results of an experiment. [file 1752-0509-4-16-S5.pdf]

The scheme to merge small networks

Because *k* networks (NWs) can be integrated one by one, in the following we demonstrate the `merging' algorithm recently integrated to GASA using two small NWs from Eq. (3) in the manuscript. Suppose that the 11 genes and two factors are involved in two small NWs, and specifically the first four equations and the last seven equations of Eq. (3) constitute these NWs. GASA is applied to two groups of genes and factors $\Gamma_1 = \{f_1, f_2, g_1, g_2, g_3, g_4\}$ and $\Gamma_2 = \{f_1, f_2, g_5, g_6, g_7, g_8, g_9, g_{10}, g_{11}\}$ to reconstruct these NWs.

The procedures are summarized as follows.

1. Calculate *SSE*/*Var* of genes in two small NWs, say Γ_1 and Γ_2 .
2. Add genes of Γ_2 one by one into Γ_1
   For a given g_i in Γ_1 (as a target gene), we add g_j from Γ_2 (where $j = 5, \ldots, 11$) one at a time into *i*-th equation in Eq.(3) as a potential regulator. For ease of implementation, a regular SA is applied to estimate coefficients of the updated *i*-th equation. Among all potential links, the link resulting in the lowest value of *SSE*/*Var* in g_i will be added to Γ_1 to form Γ'_1, and go to step 3.
3. Check network complexity
   Compute the fitness score (AIC or BIC) of the merged network Γ'_1. If the fitness score is improved, this link and its associated gene in Γ_2 are integrated into Γ_1. The *SSE*/*Var* value of gene g_i and the fitness score of Γ_1 are both updated.
4. Repeat Steps 2-3 until no other links can improve the fitness score of Γ_1.
5. Repeat the same procedures to integrate the genes of Γ_1 into Γ_2.

Table 1. The performances of GASA (with and without merging procedures) applied to the simulated NW from Eq. (3). The two small NWs were formed by the first four and last seven equations in Eq. (3), respectively. AIC and no power law restriction in the search space were applied with GASA.

Data simulated with	The inferred NW	TPR	TNR	FPR
No noise	One whole network	0.81	0.99	0.05
	One network merged from two small networks	0.75	0.95	0.25
Signal noise ratio = 4	One whole network	0.66	0.97	0.22
	One network merged from two small networks	0.69	0.94	0.28
